# Supplementary material for: Identification of IOMA-class neutralizing antibodies targeting the CD4-binding site on the HIV-1 envelope glycoprotein
Source: Nat Commun. 2022 Aug 3;13:4515. doi: 10.1038/s41467-022-32208-0 (PMC9349188; doi:10.1038/s41467-022-32208-0)
Supplement: Supplementary file 1 — Supplementary Information [file 41467_2022_32208_MOESM1_ESM.pdf]

Identification of IOMA-class neutralizing antibodies targeting the CD4-binding site on the HIV-1 envelope glycoprotein

Jelle van Schooten et al.

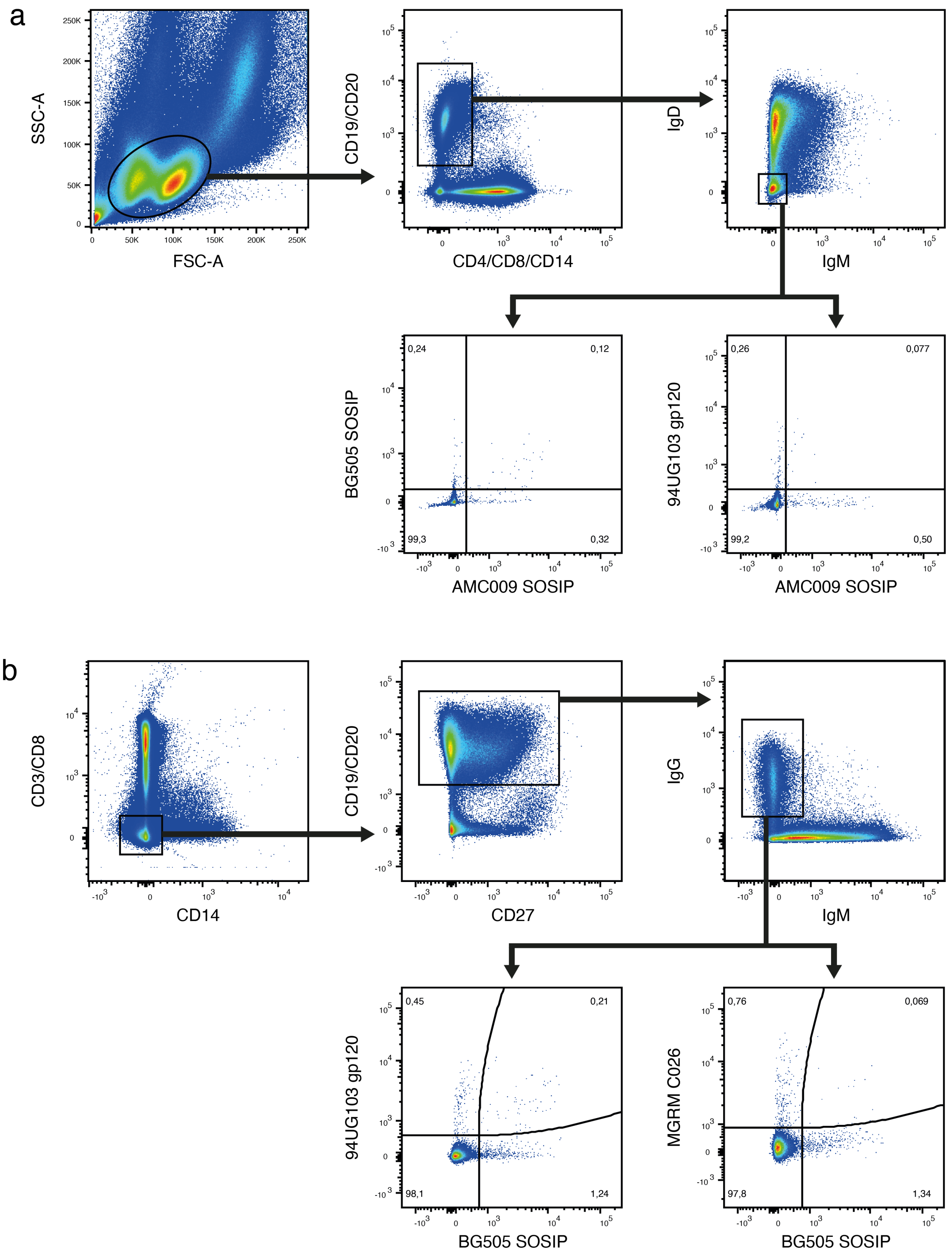

Figure S1. Sorting strategy. B cells were selected by fluorescence-activated cell sorting using fluorescently labeled Env proteins. (**a**) The gating strategy for the isolation of B cells from PBMCs taken at month 24 post-SC. (**b**) The gating strategy for the isolation of B cells from PBMCs taken at month 36 post-SC.

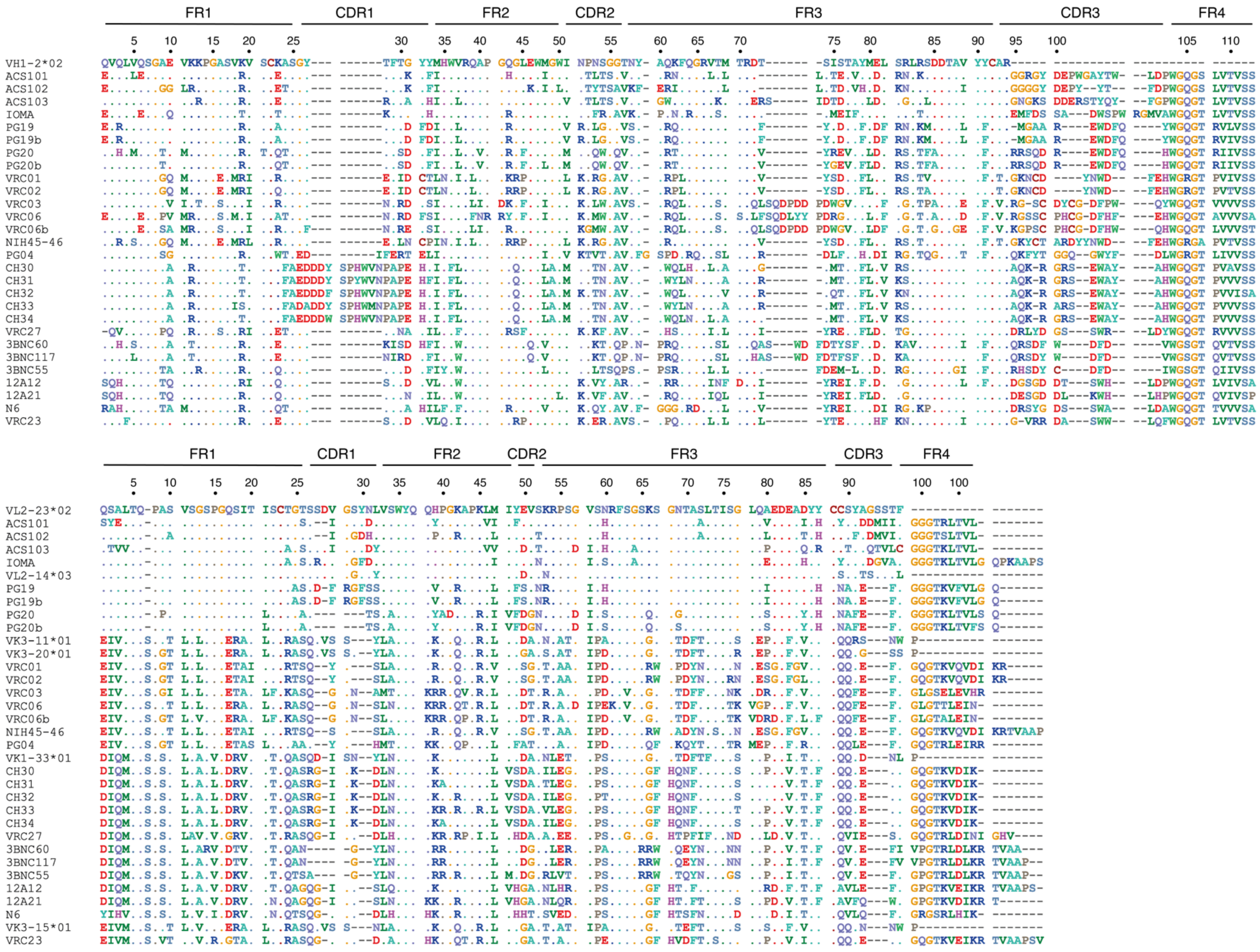

Figure S2. Comparison of heavy and light chain sequences of ACS101, ACS102 and ACS103 to germline and other VH1-2 derived bNAbs.

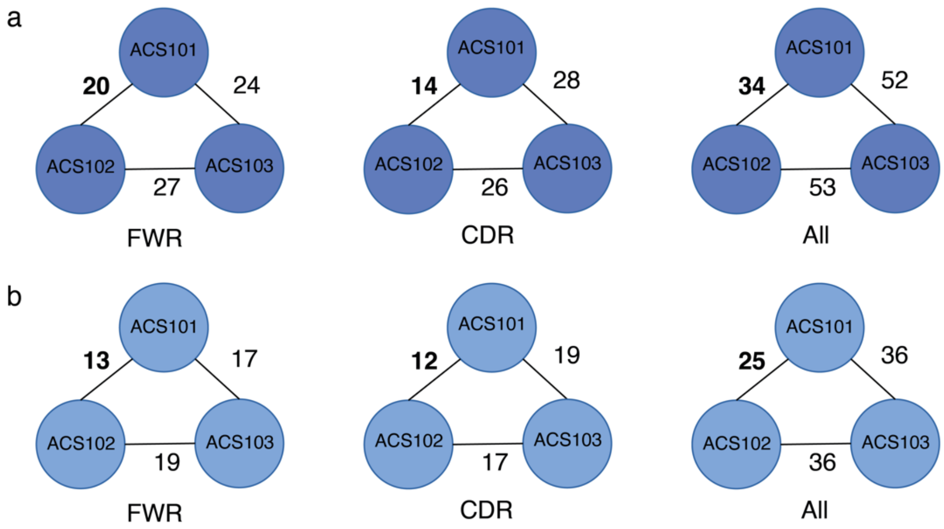

c

Reference	mAb1	mAb2	Shared mutations	% mAb1	% mAb2
IGHV1-2*02	ACS101	ACS102	32	65.3	49.2
IGHV1-2*02	ACS101	ACS103	19	38.8	35.8
IGHV1-2*02	ACS102	ACS103	18	27.7	34.0
IGHV1-2*04	ACS101	ACS102	31	64.6	48.4
IGHV1-2*04	ACS101	ACS103	18	37.5	34.6
IGHV1-2*04	ACS102	ACS103	17	26.6	32.7

Reference	mAb1	mAb2	Shared mutations	% mAb1	% mAb2
IGHD1-26*01	ACS101	ACS102	9	90.0	81.8
IGHD1-26*01	ACS101	ACS103	6	60.0	54.5
IGHD1-26*01	ACS102	ACS103	6	54.5	54.5
IGHD2-15*01	ACS101	ACS102	11	78.6	78.6
IGHD2-15*01	ACS101	ACS103	11	78.6	64.7
IGHD2-15*01	ACS102	ACS103	9	64.3	52.9

Reference	mAb1	mAb2	Shared mutations	% mAb1	% mAb2
IGHJ5*02	ACS101	ACS102	1	25.0	33.3
IGHJ5*02	ACS101	ACS103	0	0	0
IGHJ5*02	ACS102	ACS103	0	0	0

Reference	mAb1	mAb2	Shared mutations	% mAb1	% mAb2
IGLV2-23*02	ACS101	ACS102	16	44.4	50.0
IGLV2-23*02	ACS101	ACS103	10	27.8	22.7
IGLV2-23*02	ACS102	ACS103	8	25.0	18.2

Reference	mAb1	mAb2	Shared mutations	% mAb1	% mAb2
IGLJ2*01	ACS101	ACS102	4	80.0	57.1
IGLJ2*01	ACS101	ACS103	0	0	0
IGLJ2*01	ACS102	ACS103	0	0	0
IGLJ3*01	ACS101	ACS102	4	80.0	57.1
IGLJ3*01	ACS101	ACS103	0	0	0
IGLJ3*01	ACS102	ACS103	0	0	0

Figure S3. Sequence comparison of ACS101, ACS102 and ACS103. (**a**) Distance (number of aa differences) between amino acid sequences of the heavy and (**b**) light chains. The minimum distance is highlighted in bold. (**c**) Number of shared mutations between the chains compared to germline V, D and J. The percentage depicts the percentage of the shared mutations of the total mutations for the respective antibody.

a

Virus	Clade	mAbs ACS101	mAbs ACS102	mAbs ACS103	bNAb CH01-31
TRO.11	B	>50	ND	0.23	0.50
Ce1176	C	>50	ND	>50	0.15
Ce0217	C	1.3	ND	0.38	0.16
25710	C	27	ND	>50	0.36
X1632	G	1.6	ND	>50	0.12
246-F3	AC	>50	ND	>50	0.19
CNE55	CRF01_AE	37	ND	>50	0.17
BJOX2000	CRF07_BC	>50	ND	>50	15
CH119	CRF07_BC	>50	ND	>50	1.4
AMC009	B	0.28	ND	0.054	0.057

b

Virus	Clade	mAb ACS101	bNAb VRC01	bNAb CH01-31
TRO.11	B	>50	ND	0.50
TRO.11.N276Q		18	14	1.7
Ce1176	C	>50	ND	0.15
Ce1176.N276Q		11	1.4	0.058
Ce0217	C	0.80	0.32	0.11
Ce0217.N276Q		0.090	0.046	0.030
Ce0217.N279A		>50	>25	0.37
25710	C	12	0.44	0.32
25710.N276Q		>50	0.15	0.35
25710.N280D		>50	>25	1.9
X1632	G	1.5	0.15	0.094
X1632.N276Q		0.30	0.035	0.020
X1632.N280D		>50	>25	0.37
246-F3	AC	>50	ND	0.19
246-F3.N276Q		>50	0.47	1.3
CNE55	CRF01_AE	37	ND	0.17
CNE55.N276Q		0.12	0.046	0.025
BJOX2000	CRF07_BC	>50	ND	15
CH119	CRF07_BC	>50	ND	1.4
CH119.N276Q		>50	0.49	4.3
45_01dG5	B	0.023	0.020	0.030

Figure S4. Neutralization of viruses from the global panel and AMC009 by ACS101 and ACS103. (**a**) Neutralization IC_{50} (µg/ml) profile of ACS101 and ACS103 against a panel of (n=9) Tier-2 viruses and the autologous AMC009 virus. (**b**) Neutralization IC_{50} profile of ACS101 against a panel of Tier-2 viruses and their corresponding N276/N280/N279 mutants. We also assayed the 45_01dG5 virus. The bNAbs CH01-31 (an equal concentration mixture of CH01 and CH31) and VRC01 were taken along as positive controls.

Panel	Virus	Clade	mAb ACS101	bNAb CH01-31
Lasso 20 panel	9004SS-A3_4	A	>50	0.31
	Q842.d12	A	18	<0.02
	H029.12	B	23	0.47
	TRO.11	B	>50	0.50
	RPW-0510.2	B	4.2	6.5
	Ce1176	C	>50	0.15
	Ce0217	C	1.3	0.16
	25710	C	27	0.36
	7060101641_A7	C	>50	0.45
	249M_B10	C	0.81	0.12
	3728.V2.66	C	>50	0.30
	HIV-16055-2.3	C	>50	0.62
	HIV-001428_2.42	C	6.8	0.03
	25925-2.22	C	>50	1.2
	X1632	G	1.6	0.12
	246-F3	AC	>50	0.19
	CNE55	CRF01_AE	37	0.17
	BJOX2000	CRF07_BC	>50	15
	CH119	CRF07_BC	>50	1.4
	SPK_0525.13	CRF01	>50	0.22
Non-B and non-C clade virus panel	Q769.d22	A	0.36	0.10
	T250-4	AG	>50	0.07
	T928-28	AG	>50	0.44
	3016.v5.c45	D	>50	>50
	191859	D	36	4.1
	X2088-c9	G	>50	>50
	P1981-C5-3	G	>50	4.3

Panel	Virus	Clade	mAb ACS101	bNAb CH01-31
f61 virus panel	KER2008.vrc12	A	6.3	0.17
	Q259.d2.17	A	>50	7.0
	Q461.e2	AD	22	0.45
	620345.c01	CRF01_AE	>50	>25
	CNE5	CRF01_AE	>50	0.25
	0503M02138.ec1	CRF01_AE	>50	2.6
	93TH976.1	CRF01_AE	7.4	0.10
	T242-14	CRF02_AG	>50	8.5
	7165.18	B	>50	4.8
	YU2	B	0.39	0.16
	CH070.1	BC	>50	1.0
	0013095-2.11	C	ND	ND
	26191-2.48	C	>50	0.36
	3168.v4.c10	C	1.8	0.28
	ZM135M.PL10a	C	>50	2.0
	231965.c01	D	>50	3.6
	T247-23	D	>50	>25

Figure S5. Neutralization of viruses from three different panels by ACS101. Neutralization IC_{50} (μg/ml) profile of ACS101 against the Lasso 20 panel of which the nine global panel viruses are a part of, selection of viruses from the virus panel f61 and a small panel consisting of several non-B and non-C tier 2 viruses. The bNAb CH01-31 was used as positive controls.

Virus	Clade	ACS101	IOMA	VRC01
6535.3	B	>50	>50	1.7
QH0692.42	B	7.7	13	1.4
SC422661.8	B	0.65	17	0.12
PVO.4	B	1.7	0.49	0.46
TRO.11	B	>50	0.54	0.34
AC10.0.29	B	>50	>50	1.5
RHPA4259.7	B	>50	9.6	0.046
THRO4156.18	B	>50	30	3.9
REJO4541.67	B	0.41	>50	0.064
TRJO4551.58	B	3.8	12	0.089
WITO4160.33	B	>50	3.3	0.130
CAAN5342.A2	B	45	1.7	1.1
WEAU_d15_410_787	B (T/F)	0.23	3.0	0.12
1006_11_C3_1601	B (T/F)	32	0.29	0.21
1054_07_TC4_1499	B (T/F)	7.4	2.2	1.0
1056_10_TA11_1826	B (T/F)	1.1	2.1	1.1
1012_11_TC21_3257	B (T/F)	0.29	0.35	0.13
6240_08_TA5_4622	B (T/F)	5.1	4.0	0.88
6244_13_B5_4576	B (T/F)	10	0.56	0.31
62357_14_D3_4589	B (T/F)	36	11	0.98
SC05_8C11_2344	B (T/F)	4.6	1.5	0.57
Du156.12	C	>50	>50	0.092
Du172.17	C	>50	>50	>50
Du422.1	C	>50	>50	>50
ZM197M.PB7	C	1.9	>50	0.52
ZM214M.PL15	C	>50	1.7	0.82
ZM233M.PB6	C	>50	>50	2.5
ZM249M.PL1	C	0.30	>50	0.073
ZM53M.PB12	C	>50	2.3	0.87
ZM109F.PB4	C	>50	>50	0.13
ZM135M.PL10a	C	>50	>50	0.90
CAP45.2.00.G3	C	>50	>50	6.0
CAP210.2.00.E8	C	>50	>50	>50
HIV-001428-2.42	C	4.4	>50	0.019
HIV-0013095-2.11	C	>50	0.14	0.12
HIV-16055-2.3	C	>50	>50	0.10
HIV-16845-2.22	C	>50	>50	3.7
Ce1086_B2	C (T/F)	>50	0.56	0.57
Ce0393_C3	C (T/F)	0.40	15	1.0
Ce1176_A3	C (T/F)	>50	3.0	2.2

Virus	Clade	ACS101	IOMA	VRC01
Ce2010_F5	C (T/F)	>50	20	0.64
Ce0682_E4	C (T/F)	4.9	1.2	0.17
Ce1172_H1	C (T/F)	>50	>50	>50
Ce2060_G9	C (T/F)	>50	>50	0.44
Ce703010054_2A2	C (T/F)	>50	>50	0.75
BF1266.431a	C (T/F)	>50	28	0.078
246F C1G	C (T/F)	>50	>50	7
249M B10	C (T/F)	>50	18	0.29
ZM247v1(Rev-)	C (T/F)	>50	>50	0.33
7030102001E5(Rev-)	C (T/F)	17	13	1.1
1394C9G1(Rev-)	C (T/F)	4.3	0.21	0.57
Ce704809221_1B3	C (T/F)	>50	2.5	0.88
CNE19	BC	>50	>50	0.25
CNE20	BC	>50	0.044	7.0
CNE21	BC	>50	1.1	0.36
CNE17	BC	>50	>50	1.4
CNE30	BC	>50	8.3	0.82
CNE52	BC	>50	>50	0.22
CNE53	BC	1.1	0.13	0.093
CNE58	BC	>50	>50	0.16
MS208.A1	A	2.8	3.7	0.12
Q23.17	A	0.79	0.83	0.090
Q461.e2	A	10	>50	0.45
Q769.d22	A	0.12	1.4	0.028
Q259.d2.17	A	>50	>50	0.073
Q842.d12	A	4.1	2.9	0.021
0260.v5.c36	A	4.3	1.6	0.59
3415.v1.c1	A	1.7	0.71	0.090
3365.v2.c2	A	2.0	5.0	0.051
191955_A11	A (T/F)	>50	>50	0.98
191084 B7-19	A (T/F)	14	11	0.17
9004SS_A3_4	A (T/F)	>50	>50	0.51
T257-31	CRF02_AG	>50	>50	2.1
928-28	CRF02_AG	>50	>50	0.45
263-8	CRF02_AG	1.5	0.83	0.21
T250-4	CRF02_AG	>50	>50	49
T251-18	CRF02_AG	>50	>50	3.5
T278-50	CRF02_AG	>50	>50	>50
T255-34	CRF02_AG	>50	>50	0.45
211-9	CRF02_AG	>50	>50	18

Virus	Clade	ACS101	IOMA	VRC01
235-47	CRF02_AG	0.32	0.16	0.047
620345.c01	CRF01_AE	>50	>50	>50
CNE8	CRF01_AE	30	>50	0.62
C1080.c03	CRF01_AE	>50	15	1.9
R2184.c04	CRF01_AE	0.75	>50	0.084
R1166.c01	CRF01_AE	>50	8.4	2.0
R3265.c06	CRF01_AE	>50	-	0.35
C2101.c01	CRF01_AE	>50	1.5	0.20
C3347.c11	CRF01_AE	1.1	0.10	0.084
C4118.c09	CRF01_AE	>50	7.1	0.13
CNE5	CRF01_AE	>50	>50	0.32
BJOX009000.02.4	CRF01_AE	>50	>50	2.0
BJOX015000.11.5	CRF01_AE (T/F)	>50	>50	0.50
BJOX010000.06.2	CRF01_AE (T/F)	>50	>50	8.2
BJOX025000.01.1	CRF01_AE (T/F)	>50	1.5	8.7
BJOX028000.10.3	CRF01_AE (T/F)	>50	21	0.23
X1193_c1	G	3.0	>50	0.14
P0402_c2_11	G	12	16	0.17
X1254_c3	G	>50	>50	0.053
X2088_c9	G	>50	>50	>50
X2131_C1_B5	G	>50	>50	0.45
P1981_C5_3	G	>50	>50	0.36
X1632_S2_B10	G	1.2	0.64	0.11
3016.v5.c45	D	>50	>50	0.16
A07412M1.vrc12	D	>50	8.4	0.16
231965.c01	D	>50	>50	0.35
231966.c02	D	>50	2.6	0.11
6405.v4.c34	D	>50	-	-
3817.v2.c59	CD	>50	14	>50
6480.v4.c25	CD	>50	>50	0.040
6952.v1.c20	CD	>50	>50	0.057
6811.v7.c18	CD	>50	>50	0.21
89-F1_2_25	CD	>50	>50	>50
3301.v1.c24	AC	44	35	0.13
6041.v3.c23	AC	0.20	0.023	0.024
6540.v4.c1	AC	>50	>50	>50
6545.v4.c1	AC	>50	>50	>50
0815.v3.c3	ACD	24	>50	0.037
3103.v3.c10	ACD	>50	>50	1.4
MuLV	Neg. Control	>50		

Figure S6. Neutralization IC_{50} (μg/ml) of the 119-virus panel by ACS101, IOMA and VRC01. Data for IOMA and VRC01 were derived from CATNAP[1]. The bNAb VRC01 was used as a positive control,

a

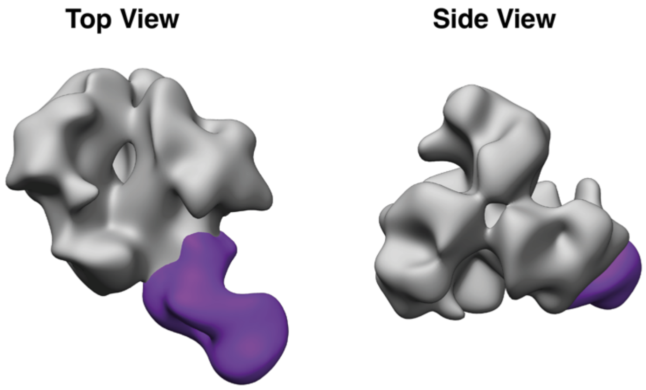

b

		mAb	bNAb
Virus	Clade	ACS124	CH01-31
25710	C	>50	0.26
TRO.11	B	>50	0.34
BJOX2000	CRF07_BC	>50	22
X1632	G	>50	0.080
Ce1176	C	>50	0.092
246-F3	AC	>50	0.16
CH119	CRF07_BC	>50	1.4
Ce0217	C	>50	0.089
CNE55	CRF01_AE	>50	0.24
AMC009	B	>50	0.036

Figure S7. ACS124 targets the gp41-gp120 interface. (**a**) ns-EM 3D reconstruction of ACS124 in complex with AMC009 SOSIP. The ACS124 Fab is colored purple. (**b**) Neutralization IC_{50} (μg/ml) profile of ACS124 against a panel of (n=9) Tier-2 viruses and the autologous AMC009 virus. The bNAb CH01-31 was used as a positive control.

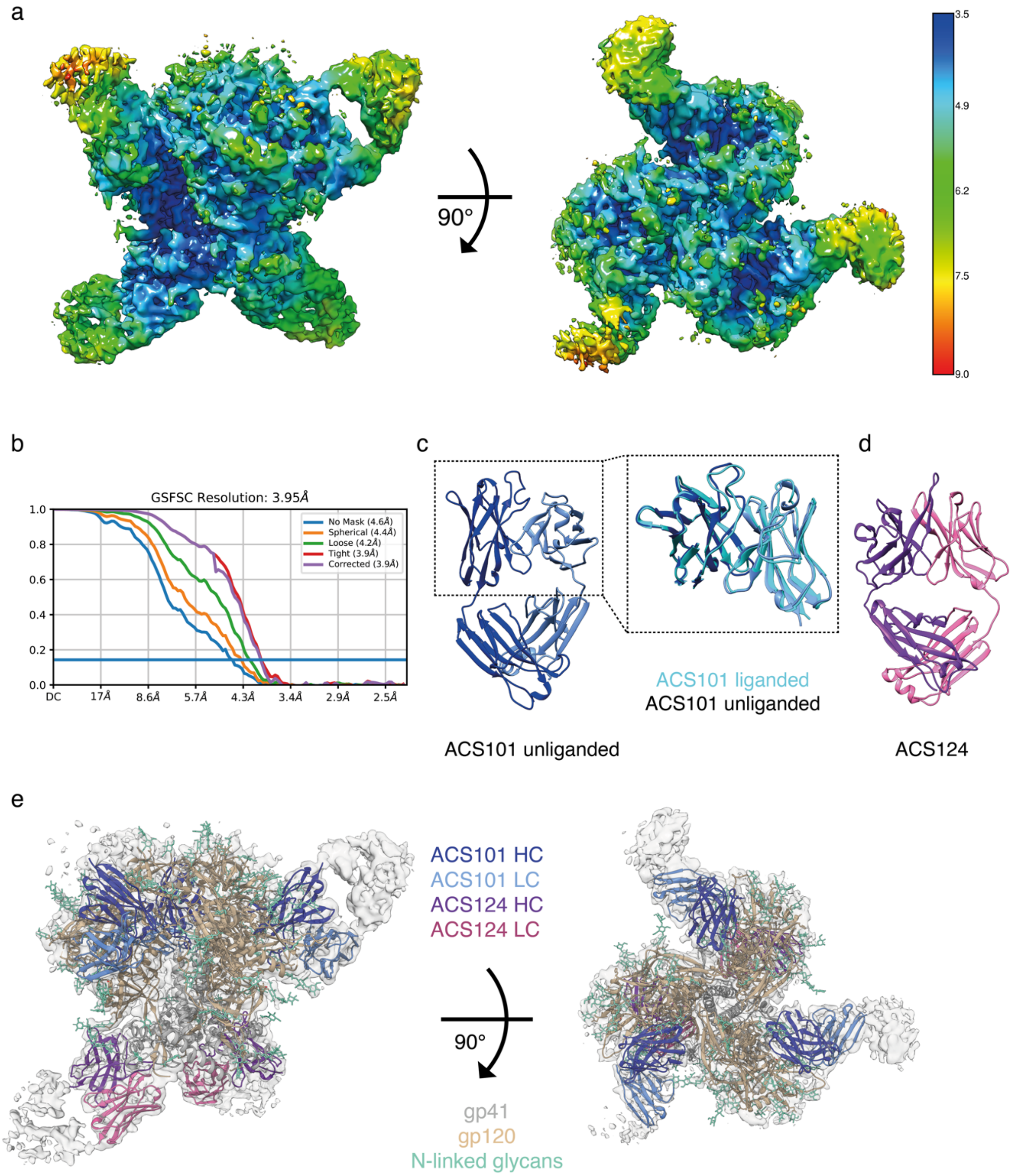

Figure S8. Cryo-EM and X-ray maps and structure determination. (**a**) Cryo-EM map of ACS101 and ACS124 Fabs in complex with AMC009 SOSIP colored by local resolution. (**b**) Gold-standard Fourier shell correlation (GSFSC) resolution estimate of ACS101 and ACS124 Fabs in complex with AMC009 SOSIP. (**c**) Crystal structure of unliganded ACS101 (HC; dark blue and LC; light blue). In the right panel the ACS101 liganded Fab is overlayed in cyan. (**d**) Crystal structure of unliganded ACS124 (HC; purple and LC; pink) Fab. (**e**) Cryo-EM map and fitted atomic model of ACS101 and ACS124 Fabs bound to AMC009 SOSIP. Only the variable domains of Fabs ACS101 and ACS124 are shown in the atomic model. The atomic model is displayed as ribbons and colored coded as indicated.

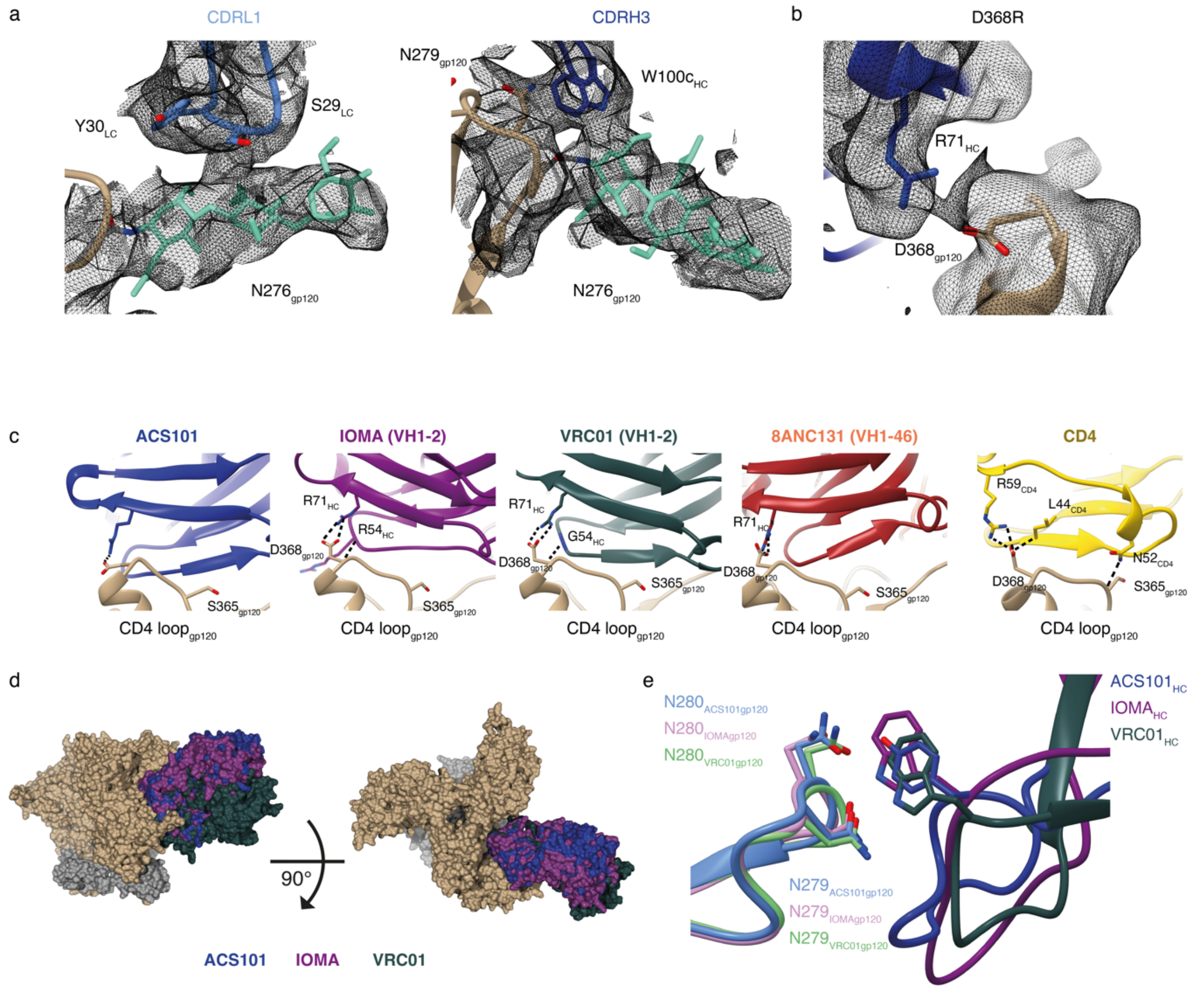

Figure S9. ACS101 interactions with the CD4bs of AMC009 SOSIP and comparison to other VH1-2 and VH1-46-derived CD4bs bNAbs. (**a**) Interaction of ACS101's CDRL1 and CDRH3 with the N276 glycan. The model is fit into the cryo-EM map (dark mesh). (**b**) Interaction of ACS101 R71$_{HC}$ with D368R. The model was fitted into the cryo-EM map (dark mesh). (**c**) Interaction of ACS101 with the CD4 loop of AMC009 SOSIP and comparison to other VH1-2-derived bNAbs IOMA (PDB:5T3Z) and VRC01 (PDB:3NGB), VH1-46-derived bNAb 8ANC131 (PDB:4RWY) and CD4 (PDB:1G9N). (**d**) Comparison of ACS101 to IOMA (PDB:5T3Z) and VRC01 (PDB:3NGB). Fabs and trimer are shown as a surface representation. Fabs are colored coded as indicated. The gp41 and gp120 subunits are depicted in grey and light brown, respectively. The structures were aligned relative to gp120. (**e**) ACS101 Y100F$_{HC}$ overlaps with VRC01 W100b$_{HC}$ (PDB:3NGB) and IOMA W100f$_{HC}$ (PDB:5T3Z). The structures were aligned relative to gp120.

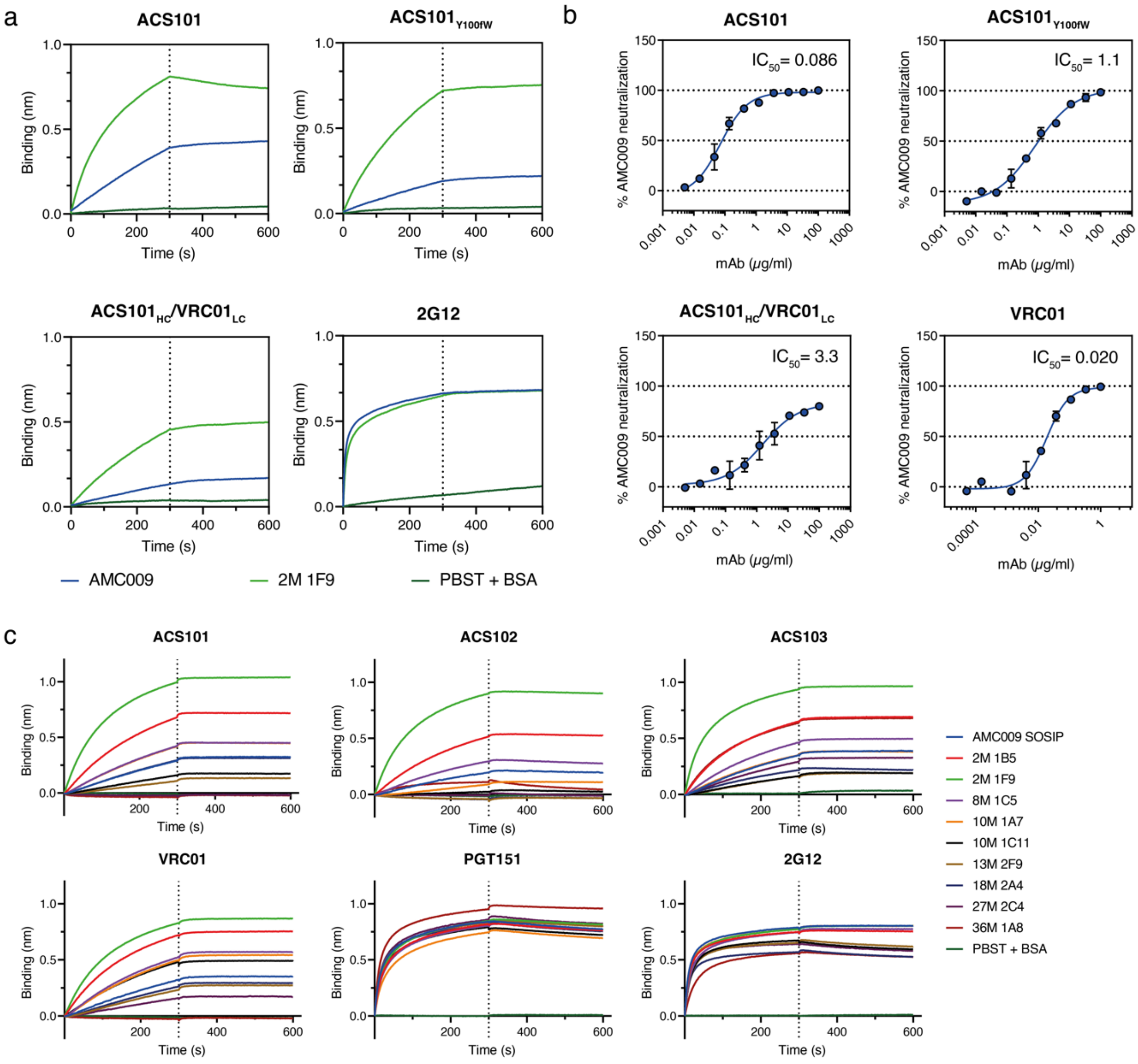

Figure S10. Bio-layer interferometry and neutralization data of ACS101 (and variants), ACS102 and ACS103 to AMC009 and longitudinal variants. (**a**) Binding of ACS101, ACS101$_{Y100fW}$ and ACS101$_{HC}$/VRC01$_{LC}$ to AMC009 and AMC009 2M 1F9 using bio-layer interferometry. The bNAb 2G12 and PBST + BSA ((PBS pH 7.4 + 0.01% (w/v) BSA + 0.002% (v/v) Tween 20) were included as a loading and negative control, respectively. (**b**) Neutralization of AMC009 by ACS101, ACS101$_{Y100fW}$ and ACS101$_{HC}$/VRC01$_{LC}$. The length of the error bars is the standard deviation at each concentration (n=2 biologically independent experiments). (**c**) Binding of mAbs to longitudinal Env using bio-layer interferometry (BLI). Binding curves are shown for ACS101, ACS102 and ACS103. The bNAbs VRC01, PGT151 and 2G12 were included as controls. PBST + BSA ((PBS pH 7.4 + 0.01% (w/v) BSA + 0.002% (v/v) Tween 20) was included as a negative control.

Map	AMC009 SOSIP.v.5.2 + ACS101 Fab + ACS124 Fab
EMDB	
**Data collection**	
Microscope	FEI Talos Arctica
Voltage (kV)	200
Detector	Gatan K2 Summit
Recording mode	Counting
Nominal magnification	36,000
Movie micrograph pixelsize (Å)	1.15
Dose rate (e^-/[(camera pixel)*s])	4.63
Number of frames per movie micrograph	56
Frame exposure time (ms)	250
Movie micrograph exposure time (s)	14
Total dose (e^-/Å^2)	49
Defocus range (μm)	-0.5 to -1.7
**EM data processing**	
Number of movie micrographs	2,364
Number of molecular projection images in map	95,062
Symmetry	C1
Map resolution (FSC 0.143; Å)	3.95
Map sharpening B-factor (Å^2)	-122
**Structure Building and Validation**	
Number of atoms in deposited model	
SOSIP gp120	10209
SOSIP gp41	2797
Fab Fv	8933
glycans	2061
MolProbity score	1.01
Clashscore	1.46
CC (mask)	0.79
d FSC model (0.5)	4.2
EMRinger score	1.88
RMSD from ideal	
Bond length (Å)	0.024
Bond angles (˚)	1.87
Ramachandran plot	
Favored (%)	97.33
Allowed (%)	2.67
Outliers (%)	0.00
Side chain rotamer outliers (%)	0.08
C-beta outliers (%)	0.00
PDB	7Z3A

Table S1. EM data collection and map/model refinement parameters.

X-ray data collection and refinement statistics		
	**ACS101**	**ACS124**
Beamline	ALS 5.0.1	SSRL 12-1
Wavelength (Å)	0.97741	0.97946
Resolution (Å)	43.75-2.31 (2.34)	33.98-1.73 (1.74)
Space group	$P3_221$	$P2_12_12_1$
Unit cell (Å)	67.13, 67.13, 199.29	62.45, 66.70, 101.93
Total reflections	237,729	556,301
Unique reflections	23,788(1113)	46,469(2300)
Multiplicity	10.0(7.1)	12.0(11.9)
Completeness (%)	100.0(100.0)	98.9(99.7)
Mean (I)/ $σ_I$	19.3(1.5)	36.3(1.3)
R_{merge}(%)	12.9(122)	15.5(179)
R_{meas}(%)	13.6(132)	16.2(187)
R_{pim}(%)	4.3(49.0)	4.6(52.6)
$CC_{1/2}$(%)	88.4(49.7)	89.4(47.2)
**Refinement**		
# Reflections used in refinement (work/free)	22,592(1134)	42,422(2018)
R_{work}(%)	21.4(30.4)	18.4(30.2)
R_{free}(%)	25.4(35.5)	22.1(39.4)
# Protein atoms (nonH)	3300	3300
# Waters	77	233
# Protein residues	431	431
RMS (bonds)	0.003	0.014
RMS (angles)	0.621	1.232
Ramachandran favored, allowed, outliers (%)	96.52, 3.48, 0.00	96.75, 3.25, 0.00
Clashscore	1.51	3.49
Wilson B (Å^2)	47	27
Average B (Å^2)	51	35

Table S2. X-ray data collection and refinement statistics. Values in parentheses correspond to the highest resolution shells.

References

1. Yoon H, Macke J, West A, et al. CATNAP: a tool to compile, analyze and tally neutralizing antibody panels. *Nucleic Acids Res*. 2015;43(W1):W213-W219. doi:10.1093/NAR/GKV404
