# Supplementary material for: Identification of IOMA-class neutralizing antibodies targeting the CD4-binding site on the HIV-1 envelope glycoprotein
Source: Nat Commun. 2022 Aug 3;13:4515. doi: 10.1038/s41467-022-32208-0 (PMC9349188; doi:10.1038/s41467-022-32208-0)
Supplement: Supplementary file 3 — Reporting Summary [file 41467_2022_32208_MOESM3_ESM.pdf]

nature portfolio

Corresponding author(s): Marit J. van Gils

Last updated by author(s): 7/10/2022

Reporting Summary

Nature Portfolio wishes to improve the reproducibility of the work that we publish. This form provides structure for consistency and transparency in reporting. For further information on Nature Portfolio policies, see our Editorial Policies and the Editorial Policy Checklist.

Statistics

For all statistical analyses, confirm that the following items are present in the figure legend, table legend, main text, or Methods section.

n/a	Confirmed	
☐	☒	The exact sample size (*n*) for each experimental group/condition, given as a discrete number and unit of measurement
☒	☐	A statement on whether measurements were taken from distinct samples or whether the same sample was measured repeatedly
☒	☐	The statistical test(s) used AND whether they are one- or two-sided<br>*Only common tests should be described solely by name; describe more complex techniques in the Methods section.*
☒	☐	A description of all covariates tested
☒	☐	A description of any assumptions or corrections, such as tests of normality and adjustment for multiple comparisons
☐	☒	A full description of the statistical parameters including central tendency (e.g. means) or other basic estimates (e.g. regression coefficient) AND variation (e.g. standard deviation) or associated estimates of uncertainty (e.g. confidence intervals)
☒	☐	For null hypothesis testing, the test statistic (e.g. *F*, *t*, *r*) with confidence intervals, effect sizes, degrees of freedom and *P* value noted<br>*Give P values as exact values whenever suitable.*
☒	☐	For Bayesian analysis, information on the choice of priors and Markov chain Monte Carlo settings
☒	☐	For hierarchical and complex designs, identification of the appropriate level for tests and full reporting of outcomes
☒	☐	Estimates of effect sizes (e.g. Cohen's *d*, Pearson's *r*), indicating how they were calculated

Our web collection on statistics for biologists contains articles on many of the points above.

Software and code

Policy information about availability of computer code

Data collection	CryoEM and crystallographic software for data colellection: Relion v3.0, Leginon, MotionCor2
Data analysis	Graphpad Prism 9.0, UCSF Chimera, Segger, cryoSPARC v3.2.0, coot, RosettaRelax, MolProbity, EMRinger, jsPISA, PDBePISA, Phaser

For manuscripts utilizing custom algorithms or software that are central to the research but not yet described in published literature, software must be made available to editors and reviewers. We strongly encourage code deposition in a community repository (e.g. GitHub). See the Nature Portfolio guidelines for submitting code & software for further information.

Data

Policy information about availability of data

All manuscripts must include a data availability statement. This statement should provide the following information, where applicable:

- Accession codes, unique identifiers, or web links for publicly available datasets
- A description of any restrictions on data availability
- For clinical datasets or third party data, please ensure that the statement adheres to our policy

Atomic coordinates and structure factors of the reported crystal structure have been deposited in the Protein Data Bank (PDB: 7U04, PDB: 7U0K). Cryo-EM reconstructions have been deposited in the Electron Microscopy Data Bank (EMD-14474) and in the Protein Data Bank (PDB: 7Z3A). The negative stain 3D EM reconstructions are deposited to the Electron Microscopy Data Bank (EMD-14475, EMD-14476, EMD-14477 and EMD-14478). The accession numbers for ACS101-103 and ACS124 BCR sequences are DDBJ/ENA/GenBank: OM744384- OM744391.

Field-specific reporting

Please select the one below that is the best fit for your research. If you are not sure, read the appropriate sections before making your selection.

☒ Life sciences ☐ Behavioural & social sciences ☐ Ecological, evolutionary & environmental sciences

For a reference copy of the document with all sections, see nature.com/documents/nr-reporting-summary-flat.pdf

Life sciences study design

All studies must disclose on these points even when the disclosure is negative.

Sample size	No sample-size calculations were performed because we only isolated antibodies from one HIV-1 infected individual.
Data exclusions	No data was excluded from analyses.
Replication	All experiments were performed in duplicate and replication was successful.
Randomization	Not relevant for this study because we describe the structural characterization of an antibody bound to its antigen that was isolated from one HIV-1 infected individual.
Blinding	Not relevant for this study because we describe the structural characterization of an antibody bound to its antigen which was isolated from one HIV-1 infected individual

Reporting for specific materials, systems and methods

We require information from authors about some types of materials, experimental systems and methods used in many studies. Here, indicate whether each material, system or method listed is relevant to your study. If you are not sure if a list item applies to your research, read the appropriate section before selecting a response.

Materials & experimental systems

n/a	Involved in the study	
☐	☒	Antibodies
☐	☒	Eukaryotic cell lines
☒	☐	Palaeontology and archaeology
☒	☐	Animals and other organisms
☐	☒	Human research participants
☒	☐	Clinical data
☒	☐	Dual use research of concern

Methods

n/a	Involved in the study	
☒	☐	ChIP-seq
☒	☐	Flow cytometry
☒	☐	MRI-based neuroimaging

Antibodies

Antibodies used	The ACS101, ACS102, ACS103 and ACS124 (including the various mutants) antibodies were generated by ourselves. All the other antibodies used were provided by the NIH AIDS Research and Reference Reagent Program (VRC01, 2G12, PGT151, CH01-31). All antibody dilutions used are given in either the figures and also described in the method section of the manuscript.
Validation	N.A.

Eukaryotic cell lines

Policy information about cell lines

Cell line source(s)	HEK293F ((Invitrogen, cat no. R79009) and TZM-bl (JC57BL-13) cell lines
Authentication	None of the cell lines were validated.
Mycoplasma contamination	All cell lines were tested negative for mycoplasma contamination.
Commonly misidentified lines (See ICLAC register)	No commonly misidentified lines were used in this study.

Human research participants

Policy information about studies involving human research participants

Population characteristics	PBMCs were obtained from a male participant, donor H18877, who was enrolled in the MSM (men having sex with men) component of the Amsterdam Cohort Studies on HIV/AIDS (this data has been published before see methods section of the manuscript). Individual H18877 was infected with a subtype B HIV-1 variant and seroconverted during active follow-up. He did not receive combination antiretroviral therapy during the period when PBMCs and/or sera were collected. During this period, he had a detectable viral load and his CD4 T cell count was stable. In addition, H18877 was homozygous for the CCR5 gene and had no protective HLA type. The longitudinal development of the HIV-1-specific neutralizing activity in individual H18877 has been previously reported, and H18877 was assigned there as individual C1 (see methods).
Recruitment	This data has been published previously, please see methods section for the correct references.
Ethics oversight	This data has been published previously, please see the methods section for the correct references. The Amsterdam Cohort Studies are conducted in accordance with the ethical principles set out in the Declaration of Helsinki, and written consent was obtained prior to data collection. The study was approved by the Academic Medical Center Institutional Medical Ethics Committee.

Note that full information on the approval of the study protocol must also be provided in the manuscript.
